# Genome Sequence Analysis Reveals Evidence of Quorum-Sensing Genes Present in *Aeromonas hydrophila* strain KOR1, Isolated from a Mangrove Plant (*Kandelia obovata*)

**DOI:** 10.1128/genomeA.01461-15

**Published:** 2015-12-10

**Authors:** Mengqing Yin, Zhiping Ma, Zhonghua Cai, Guanghui Lin, Jin Zhou

**Affiliations:** a Center for Earth System Science, Tsinghua University, Beijing, China; b Ocean Science and Technology Division, Graduate School at Shenzhen, Tsinghua University, Shenzhen, China

## Abstract

*Aeromonas hydrophila* strain KOR1, isolated from mangrove rhizosphere soil, has the ability to produce the quorum-sensing signal molecule. Here, we report the 4.78-Mb genome sequence of strain KOR1, and found its quorum-sensing encoding gene *LuxR*. The data will be crucial to understanding the quorum-sensing-dependent phenotypes of this bacterium.

## GENOME ANNOUNCEMENT

There has been increased interest in quorum sensing (e.g., *N*-acylhomoserine lactone [AHL] molecules) to evaluate the interactions between microbes and mangrove plants (1). In the rhizosphere environment, previous studies have shown that root-associated bacterial isolates produce AHLs and exhibit various functions, including facilitating nitrogen fixation (2), promoting plant growth (3), stimulating primary root elongation (4), and resisting pathogens (5). Recently, we isolated *Aeromonas hydrophila* KOR1 (a nitrogen-fixing bacterium) from *Kandelia obovata*, and found that it can secrete short-chain (C_4_–C_6_) AHL molecules. Although the phenotype was observed, this type of functionality has not yet been elucidated at the genomic level. Moreover, no genome data of this strain from a wetland environment have been reported. Here, we performed whole-genome sequencing of this bacterium and searched for its AHL-encoding gene.

The genomic DNA of *A. hydrophila* KOR1 was extracted using the DNA extraction kit (MoBio, USA) according to the manufacturer’s instructions. The whole-genome shotgun project of *A. hydrophila* KOR1 was performed using pair-end sequencing in an Illumina MiSeq sequencing platform (Illumina, USA), which was performed by Shenzhen Hengchuan Gene-Tech. Co., Ltd. The reads were assembled with SOAPdenovo version 2.04 (6), and the sequence was annotated using the RAST annotation server (7). tRNA and rRNA genes were predicted by tRNAscan-SE (8) and RNAmmer (9), respectively. Genes were predicted using Glimmer version 3.02 (10) and were annotated by searching against the NCBI-NR and KEGG databases.

The whole genome comprised 5 scaffolds consisting of 14 contigs with a total length of 4,783,170 bp. The G+C content was 61.82%. From the draft genome sequence, a total of 4,249 genes were identified in the genome with an average length of 956 bp. Homologous comparison by BLAST found 2,833 coding sequences involving 22 functional COG groups and a portion of the coding sequences involving 34 metabolic pathway KEGG groups. A total of 149 noncoding RNAs were found in the genome, including 113 tRNAs, 30 rRNAs, and 6 sRNAs.

Based on the functional categories of COG (http://www.ncbi.nlm.nih.gov/COG), 180 genes were found to be related to signal transport and interaction processing. Potentially, these genes are key features of *A. hydrophila* KOR1 that enable it to release or receive all kinds of signals, including biological and chemical information. With respect to AHL signals, the AHL-encoding gene (*LuxR*) was predicted to be located at contig 8, with a length of 623 bp. In addition, a putative autoinducer-2 production protein *LuxS* gene was also found. The whole-genome analysis revealed the presence of a quorum-sensing encoding gene (*LuxR*), which is crucial for understanding the *A. hydrophila* KOR1 AHL-dependent phenotypes and their ecological function in the rhizosphere environment.

### Nucleotide sequence accession number.

The complete annotated genome sequence was deposited in GenBank with the accession number LJOE00000000.
